# Subspecialty surveillance of long-term course of small and moderate muscular ventricular septal defect: heterogenous practices, low yield

**DOI:** 10.1186/1471-2431-14-282

**Published:** 2014-11-04

**Authors:** Erik L Frandsen, Aswathy V House, Yunbin Xiao, David A Danford, Shelby Kutty

**Affiliations:** Division of Pediatric Cardiology, University of Nebraska College of Medicine and Children’s Hospital and Medical Center, 8200 Dodge St, Omaha, NE 68114 USA

**Keywords:** Congenital heart disease, Muscular ventricular septal defect, Echocardiography, Follow-up practices

## Abstract

**Background:**

No expert consensus guides practice for intensity of ongoing pediatric cardiology surveillance of hemodynamically insignificant small and moderate muscular ventricular septal defect (mVSD). Therefore, despite the well-established benign natural history of mVSD, there is potential for widely divergent follow up practices. The purpose of this investigation was to evaluate (1) variations in follow up of mVSD within an academic children’s hospital based pediatric cardiology practice, and (2) the frequency of active medical or surgical management resulting from follow up of mVSD.

**Methods:**

We retrospectively reviewed records of 600 patients with isolated mVSD echocardiographically diagnosed between 2006 and 2012. Large mVSD were excluded (n = 4). Patient age, gender, echocardiographic findings, provider, recommendations for follow up, and medical and surgical management were tabulated at initial and follow up visits. Independent associations with follow up recommendations were sought using multivariate analysis.

**Results:**

Initial echocardiography showed small single mVSD in 509 (85%), multiple small mVSD in 60 (10%), and small-to-moderate or moderate single mVSD in 31 (5%). The mean age at diagnosis was 15.9 months (0–18.5 years) and 25.7 months (0–18.5 years) at last follow up. There was slight female predominance (56.3%). Fourteen pediatric cardiology providers recommended 316 follow up visits, 259 of which were actually accomplished. There were 37 other unplanned follow up visits. No medical or surgical management changes were associated with any of the follow up visits. The proportion of patients for whom follow up was advised varied among providers from 11 to 100%. Independent associations with recommendation for follow up were limited to the identity and clinical volume of the provider, age of the patient, and the presence of multiple, small-to-moderate, or moderate mVSD.

**Conclusions:**

In this large series of moderate or smaller mVSD, pediatric cardiology follow up was commonly recommended but resulted in no active medical or surgical management. Major provider based inconsistency in intensity of follow up of mVSD was identified, but is difficult to justify.

## Background

Ventricular septal defect (VSD) is the most common isolated congenital cardiac defect, representing up to 40% of congenital cardiac defects diagnosed in infancy [1]. Defects located within the muscular septum constitute the more frequently seen type of VSD [2]. The natural history of muscular ventricular septal defects (mVSD) is well described. Up to 76% undergo spontaneous closure by the end of the first year of life [3–7], a large proportion of which close by six months of age [3, 4, 8]. The rates of spontaneous closure are higher for mVSD compared to membranous VSD [6, 7]. In a series of apical mVSD’s diagnosed between 1 day and 13 years of age, up to 44% spontaneous closure rate was reported within 3 years of diagnosis [4]. A 1.8% risk of infective endocarditis has been reported for VSD, mostly occurring in the perimembranous type [9]. Neumayer et al. reported no endocarditis complication for isolated mVSDs in their series [10]. The majority of small mVSD do not require surgical management, and for those small defects that remain patent, long-term complication rates are minimal [9–12].

Despite the well-described natural history and benign course, no expert consensus guides practice for intensity of ongoing pediatric cardiology surveillance of small mVSD. A survey by Smith and Qureshi demonstrates the general divergence of opinion regarding follow up for congenital heart defects [13]. Follow up practice patterns of hemodynamically insignificant mVSD have not been specifically studied previously. The purpose of this investigation is to evaluate (1) variations in follow up of mVSD within an academic children’s hospital based group pediatric cardiology practice, and (2) the frequency of active medical or surgical management resulting from follow up of mVSD.

## Methods

This was a retrospective cohort study performed in a university affiliated academic children’s hospital, in accordance with the ethical standards laid down in the 1964 Declaration of Helsinki and its later amendments. The Institutional Review Board of the University of Nebraska Medical Center approved the study. Informed consent was waived for subjects included. Pediatric cardiology databases were used to identify patients who had echocardiographic diagnosis of mVSD between 2006 and 2012.

In accordance with the recommended standards and guidelines for pediatric echocardiography set by the Intersocietal Accreditation Commission for Echocardiography Laboratories (ICAEL) and the American Society of Echocardiography, echocardiography was performed by registered cardiac sonographers, and reported by board certified pediatric cardiologists. Only those patients who had hemodynamically insignificant mVSD on echocardiography, evidenced by restrictive left to right shunting, absence of ventricular hypertrophy, and normal pulmonary artery pressure were included. Patients with age appropriate patent foramen ovale and patent ductus arteriosus were also included. Specific exclusion criteria consisted of (1) patients with additional VSD located in areas besides the muscular septum, (2) patients with any associated cardiac lesion, (3) patients with large mVSD, and 4) patients with previous cardiothoracic surgery.

Patient age, gender, echocardiographic findings, PC provider, recommendations for follow up, and medical and surgical management at initial and follow up visits were obtained from medical records review. Patients were categorized based on echocardiographic reported size of mVSD (small, small-to-moderate, moderate, large) and number of mVSD (isolated or multiple) at initial diagnosis. Patient age was categorized as younger (<3 months at entry into study) vs. older. Pediatric cardiology providers were categorized based on training (physician pediatric cardiologist vs. physician assistant), on clinical volume during the study (≥50 cases vs. < 50 cases), and on echocardiographic expertise (official interpreter of echocardiograms vs. non-interpreter).

### Follow-up

At initial visit and each subsequent follow up, pediatric cardiology provider visit records were reviewed. The following information was obtained from the records for each provider: (1) continued presence or spontaneous closure of mVSD by clinical exam and by echocardiography, (2) if the mVSD remained open, provider impression regarding hemodynamic effects of the defect (significant or insignificant), (3) recommendation for follow up, (4) time until next recommended follow up, and (5) recommendation for medical or surgical management.

### Statistics

Descriptive statistics for categorical variables are reported as frequency and percentage. Univariate comparisons of outcome were made among dichotomous variables using the Chi square test. Candidate independent variables were selected based on univariate correlation (p < 0.15), and were incorporated into a multivariate logistic regression to generate a model associating them with follow up recommendation. Alpha to enter and alpha to exclude variables from the stepwise process were both 0.15. A p value of <0.05 represented significance. Statistical analysis was performed with commercially available computer software (Minitab 16.0, Minitab Inc., State College, PA).

## Results

### Patient characteristics

After exclusion of 4 patients with large mVSD, the study population consisted of 600 patients with mVSD. None of the patients had a swiss cheese septum. There were 262 males (43.7%) and 338 females (56.3%). The mean age at diagnosis was 15.9 months (0–18.5 years) and at last follow up 25.7 months (0–18.5 years). Initial Echo showed isolated small mVSD in 509 patients (85%), multiple small mVSD in 60 (10%), isolated small-to-moderate mVSD in 12 (2%), and isolated moderate mVSD in 19 (3%) patients (Table 1).

**Table 1:** **Characteristics of muscular VSD and associated rate of follow up recommendation**

mVSD size	Number of patients	Rate of follow up recommendation (%)
Isolated small	509 (85%)	32
Isolated small-to-moderate	12 (2%)	42
Isolated moderate	19 (3%)	84
Multiple small	60 (10%)	58

*mVSD* muscular ventricular septal defect.

### Follow-up patterns

In all, 316 follow up visits were recommended by fourteen pediatric cardiology providers, of which 259 were actually accomplished. There were 37 other unplanned follow up visits. No recommendations for medical or surgical management were made at any of the follow up visits. The mean follow up duration was 1.4 years (range 1 month to 5 years). For those patients who were dismissed from follow up, the mean interval from initial visit to dismissal was 0.82 years (range 0 days to 12.3 years). There were 12 MD (evaluated 85% of small mVSDs) and 2 non-MD physician assistant providers (evaluated 15% of small mVSDs).

Follow up was recommended for patients with an isolated small mVSD at a rate of 32%, versus 68% for small-to-moderate and moderate isolated mVSD, and 58% for multiple small mVSD (p < 0.001, Table 1). Younger age at first visit was associated with greater rate of recommendation for follow up. The mean age at first visit for patients who were recommended follow up was 11.4 months vs. 18.4 months for patients who were not recommended follow up (p = 0.006).

There was significant variability in follow up recommendations between providers, ranging from 11-100% (Table 2). Independent associations with recommendation for follow up were limited to high clinical volume provider, the mVSD characteristics (multiple mVSD, isolated small-to-moderate, or isolated moderate mVSD), and patient age under 3 months at first visit (Table 3). Patient gender, echo-reading provider, and non-MD provider did not appear to be independently related to subspecialty follow-up recommendations (Table 3).

**Table 2:** **Patient characteristics and pediatric cardiology provider follow up practice patterns for small muscular VSD**

Pediatric cardiology provider	Number of patients for each provider	Number of patients with small mVSD	Percentage of patients with small mVSD	Number of follow up recommendations	Percentage of patients for which follow up was recommended
1	4	4	100	4	100
2	274	244	99	31	11
3	27	22	82	24	89
4	83	72	87	27	33
5	6	5	83	2	33
6	87	69	79	59	68
7	38	28	74	16	42
8	5	4	80	3	60
9	11	8	73	8	73
10	13	11	85	3	23
11	23	18	78	21	91
12	18	13	72	17	94
13	6	6	100	4	67
14	5	5	100	1	20

*mVSD* muscular ventricular septal defect.

**Table 3:** **Independent associations with recommendation for follow up**

	Univariate analysis			Multivariate analysis	
Variable	N _with_(% Follow-up recommended)	N _without_(% Follow-up recommended)	p	Odds ratio (95% CL)	p
High volume provider	444 (26.3)	156 (66.0)	<0.001	5.23 (3.44-7.93)	<0.001
Non MD provider	88 (31.8)	512 (37.5)	0.303	--------	-----
Echo reader provider	451 (33.7)	149 (45.6)	0.009	1.20 (0.78-1.84)	0.409
Male gender	262 (36.3)	338 (37.0)	0.855	--------	-----
Multiple mVSD	60 (58.3)	540 (34.3)	<0.001	0.38 (0.21-0.68)	0.001
Small-moderate or moderate mVSD	31 (67.7)	569 (35.0)	<0.001	0.27 (0.12-0.63)	0.002
Age < 3 months	304 (44.1)	296 (29.0)	<0.001	0.57 (0.39-0.83)	0.003

## Discussion

No clear consensus exists about the value of follow up for small mVSD [14]. Of 52 respondents to a small survey conducted among pediatric cardiology providers in the UK, the majority would follow up a hemodynamically insignificant VSD in three years, 15% would do so in one year, and less than 10% would dismiss the patient upon diagnosis [13]. Our experience confirmed the striking variability in actual practice that the survey would lead us to expect. In this report, individual providers recommended pediatric cardiology follow up for as few as 11% and as many as 100% of their patients.

Small mVSD are common. Studies prior to the routine use of echocardiography determined an incidence of 1.3-3.3 VSD per 1000 live births [15, 16], but with the advent of echocardiography, incidence has increased significantly [8, 17, 18]. Because the population of children with small mVSD is large, a policy of routine pediatric cardiology follow up would consume substantial resources in the subspecialty outpatient clinic. The benefits of this investment are not obvious; given the natural history studies that show uncomplicated small mVSD carries a high likelihood of spontaneous closure [3–7, 11, 12], and little if any prospect for clinical deterioration [11, 12]. It is impressive that the provider with the largest practice had the lowest follow up rate of 11%, while providers with the smaller clinical practice had the higher rates. This would suggest that provider experience might be associated with better resource utilization, perhaps driven by greater confidence.

Our data confirms that among a large number of pediatric cardiology follow up visits for mVSD, we could identify no case in which it resulted in active medical or surgical management of the condition. Certain clinical features, such as younger age of the patient, number of defects, and any suggestion that the defect may be moderate size, appeared to influence the likelihood that follow up would be arranged without identifying a subgroup in which active medical or surgical management actually took place. Patients with multiple small mVSD in this series were recommended follow up more often than isolated small mVSD. Patients with isolated moderate sized mVSD were recommended follow up at a greater rate than those with isolated small-to-moderate and small sized defects. We speculate that perceived differences in spontaneous closure rates (between defects of different sizes and numbers) might have influenced follow up recommendations. It is known that age at diagnosis and method of diagnosis influence reported spontaneous closure rates for VSD [3, 6, 7, 19, 20]. Young age at diagnosis was a significant predictor of spontaneous closure [19, 21].

Even after accounting for age of the patient and perceived size and number of muscular VSD’s, we found that follow-up recommendations are highly provider dependent. We interpret this as a sign that follow up recommendations for mVSD in our series were not evidence-based. Recently, the implementation of standardized clinical assessment management plans (SCAMPS) methodology reduced resource use and practice variation in the outpatient evaluation of pediatric cardiology chest pain [22]. A similar process could be applied to establish care standards for subspecialty surveillance of small mVSD.

Living in a society that presumes that more healthcare is better healthcare, and supposing that any cardiac defect represents a threat, concerned parents may exert pressure on pediatric cardiologists to provide ongoing subspecialty care for small mVSD. No physical harm likely arises from asking these patients to return for subspecialty follow-up, but because there were no interventions for those in this series who did return, it is intriguing to speculate whether harm of other sorts might be done. Pediatric cardiologists have long been sensitive to concerns that if benign conditions are permitted to be understood as threats, that inappropriate restrictions might be imposed on the patients by well-meaning parents, other care providers, or school personnel [23]. It is beyond the scope of this investigation to decide if diligent subspecialty follow-up for small mVSD might foster consequences akin to ‘cardiac nondisease’ described years ago for innocent murmur [23, 24]. High clinical volume providers in this series may have already concluded based on experience that for the vast majority of small mVSD, the costs and potential for adverse psychological impact associated with routine subspecialty surveillance outweigh any benefits.

### Limitations

This study has the limitations associated with a retrospective cohort study. Several other factors may have impacted follow up patterns on a case-by-case basis. This type of investigation does not account for everything that plays into pediatric cardiology provider decision to recommend follow up. Neither does it provide timelines for follow up of small hemodynamically insignificant mVSDs. Clinical scenarios for each patient may be different, which influences pediatric cardiology provider decisions.

## Conclusion

Wide practice variation was observed in the surveillance frequency for small and moderate sized mVSD within a 14-provider pediatric cardiology group, in the absence of active medical or surgical management. Allowances should always be made permitting practice variations in exceptional cases, so an occasional mVSD will receive follow up, but this investigation does not support a practice of regularly scheduled subspecialty surveillance.

## Abbreviations

mVSD: muscular ventricular septal defect.
